# Prenatal programming: adverse cardiac programming by gestational testosterone excess

**DOI:** 10.1038/srep28335

**Published:** 2016-06-22

**Authors:** Arpita K. Vyas, Vanessa Hoang, Vasantha Padmanabhan, Ebony Gilbreath, Kristy A. Mietelka

**Affiliations:** 1 Department of Pediatrics and Human Development, Michigan State University, E. Lansing, MI, 48824, USA; 2 Department of Pediatrics, University of Michigan, Ann Arbor, MI, 48109, USA; 3 Pathobiology Department, College of Veterinary Medicine, Nursing and Allied Health, Tuskegee University, Tuskegee, AL, 36088, USA; 4 Pathobiology and Diagnostic Investigation, College of Veterinary Medicine, Michigan State University, USA.

## Abstract

Adverse events during the prenatal and early postnatal period of life are associated with development of cardiovascular disease in adulthood. Prenatal exposure to excess testosterone (T) in sheep induces adverse reproductive and metabolic programming leading to polycystic ovarian syndrome, insulin resistance and hypertension in the female offspring. We hypothesized that prenatal T excess disrupts insulin signaling in the cardiac left ventricle leading to adverse cardiac programming. Left ventricular tissues were obtained from 2-year-old female sheep treated prenatally with T or oil (control) from days 30–90 of gestation. Molecular markers of insulin signaling and cardiac hypertrophy were analyzed. Prenatal T excess increased the gene expression of molecular markers involved in insulin signaling and those associated with cardiac hypertrophy and stress including insulin receptor substrate-1 (IRS-1), phosphatidyl inositol-3 kinase (PI3K), Mammalian target of rapamycin complex 1 (mTORC1), nuclear factor of activated T cells –c3 (NFATc3), and brain natriuretic peptide (BNP) compared to controls. Furthermore, prenatal T excess increased the phosphorylation of PI3K, AKT and mTOR. Myocardial disarray (multifocal) and increase in cardiomyocyte diameter was evident on histological investigation in T-treated females. These findings support adverse left ventricular remodeling by prenatal T excess.

Organisms experiencing adverse intrauterine environment during a critical period of development have alterations at the molecular and gene expression level that start the process of increased susceptibility to adult disease and reduced ability to adapt to insult. It is well established that suboptimal intrauterine environments leads to increased incidence of cardiovascular disease^1^. One such suboptimal intrauterine environment is altered hormonal milieu that has been shown to lead to intrauterine programming of several organs of the growing fetus^2^. For instance, testosterone (T) excess *in-utero*, during critical windows of development, leads to adverse reproductive and metabolic programming in several animal models^3^,^4^. Many of these perturbations recapitulate the features seen in polycystic ovarian syndrome (PCOS), a common endocrine disorder in women of child-bearing age with a prevalence rate of 5–8%^5^. The exact etiology of PCOS is not clear but there is evidence that prenatal environment could play a significant role^3^. The clinical manifestations of the condition include hyperandrogenism, oligo-/anovulation, and multifollicular ovarian morphology^5^,^6^. Metabolic disturbances are highly prevalent in this population with insulin resistance in 50–70% of the patients^7^, impaired glucose tolerance in 40%, and type 2 diabetes in 10% of the patients^8^,^9^.

Women with PCOS are also at increased risk of cardiovascular disease and myocardial infarction^10^,^11^. They have higher incidences of hypertension, coronary artery disease and diastolic dysfunction^12^. Available evidence points to early onset of cardiac remodeling resulting in increased left ventricular mass in young women with PCOS^13^, and reduced left ventricular ejection fraction and diastolic dysfunction in older women with PCOS^12^. The underlying mechanisms leading to cardiovascular compromise in PCOS are unclear. Animal models of PCOS provide a unique opportunity to investigate underlying mechanisms. Prenatal T-treatment also causes hypertension in rats and sheep^14^,^15^, supportive of prenatal programming of vascular disease, and likely adverse cardiac programming. Because prenatal T excess also induces intrauterine growth restriction (IUGR) and metabolic perturbations such as insulin resistance in sheep^4^,^16^, attributes that are closely linked to adverse cardiovascular effects in humans^17^, any adverse cardiac programming in prenatally T-treated sheep may be the indirect consequence of IUGR and the insulin resistance induced by prenatal T excess^16^,^18^. Alternatively, effects of prenatal T excess may also be mediated by direct actions of androgen. Androgen receptors are present in cardiac tissue^19^ and T can induce cardiac hypertrophy both *in-vitro* and *in-vivo*^20^,^21^. Prenatally T-treated sheep are functionally hyperandrogenic^4^,^22^.

A number of molecular mediators involved in insulin signaling such as phosphatidyl inositol-3 kinase (PI3K)/protein kinase B (AKT)/mammalian target of rapamycin complex 1 (mTORC1) signaling pathway have been implicated in cardiac hypertrophy^23^,^24^. Evidence from *in-vitro*^25^ studies indicates that T triggers cardiac hypertrophy by activation of mTORC1 pathway. Considering that prenatal T induces functional hyperandrogenism and insulin resistance in sheep^4^,^18^, alters insulin signaling in liver and muscle^26^, and both T and insulin have the potential to alter cardiac function, we hypothesized that prenatal T excess also disrupts insulin signaling and upregulates molecular markers of cardiac hypertrophy leading to adverse cardiac remodeling.

## Results

Body weights (kg; Mean ± SEM) were not different between control and T-treated sheep at the time of harvest (T-treated 69.4 ± 5.4 vs. control 63.09 ± 4.9 p = 0.40, cohen’s d = 0.44). Fasting insulin levels (IU/ml; Mean ± SEM) of prenatally T-treated females trended to be higher (T-treated 15.7 ± 4.7 vs. control 9.23 ± 2.12, p = 0.26), with Cohens analysis revealing a modest increase (d = 0.65). Fasting glucose (mg/dl; Mean ± SEM) levels averaged 58 ± 3.9 and 52 ± 1.7 for the T-treated females and controls, respectively (p = 0.28), with Cohens analyses revealing a modest effect size difference (d = 0.67). Insulin to glucose ratio also tended to be higher in the T-treated group (0.25 ± 0.06) compared to the control group (0.17 ± 0.04), with Cohen’s analyses showing a modest increase (d = 0.60). Left ventricular weights of the T-treated females also trended higher but did not differ statistically from control sheep (T-treated 164.9 ± 9.9 grams vs control 152 ± 7.2 grams, p = 0.30, cohen’s d = 0.54).

### Mediators of insulin signaling in left ventricular tissue

**Figure 1:**
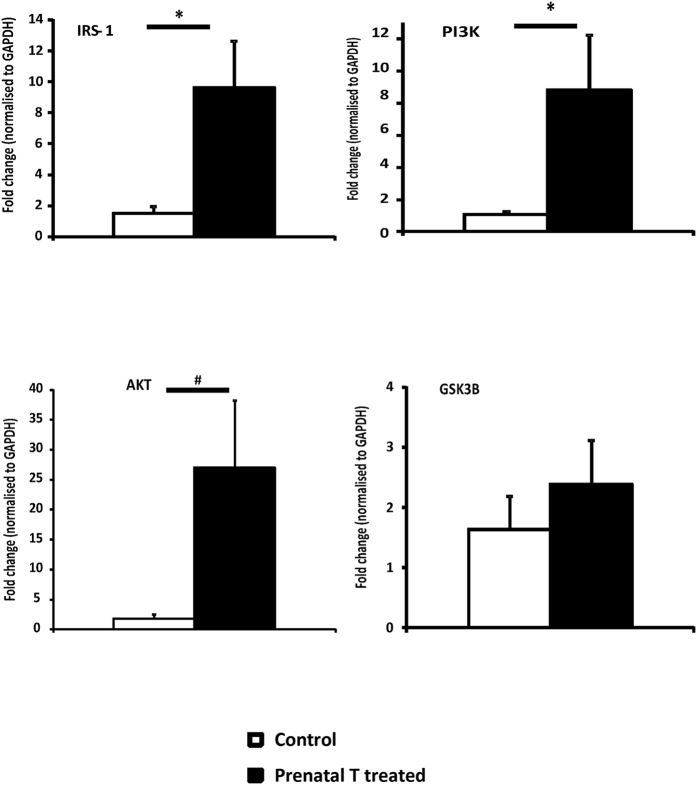
Gene expression (Mean ± SEM) of proteins involved in insulin signaling pathway measured by qRT-PCR in left ventricle tissue obtained at 2 year of age (n = 6–8 per group). Two tail student t-test was used to test differences between T-treated and control and * assigned to p < 0.05 and ^#^ for p value 0.06. The Cohen’s effect size analysis revealed large differences between T-treated and control group for genes involved in insulin signaling pathways (Cohen’s d: AKT = 1.11, IRS-1 = 1.33, PI3K = 1.29, GSK3b = 0.452). Abbreviations: IRS-1 insulin receptor substrate 1, PI3K Phosphoinositide 3-kinase, Akt protein kinase B, GSK3B Glycogen synthase kinase 3 beta.

A significant increase in gene expression of proteins involved in the insulin signaling pathway was found in the left ventricle of prenatally T-treated females compared to controls. Prenatal T excess increased the expression level of IRS-1 by 6.4 fold (p = 0.027), PI3K by 8.5 fold (p = 0.04), and AKT by 27 fold (p = 0.06), compared to control (Fig. 1).

**Figure 2:**
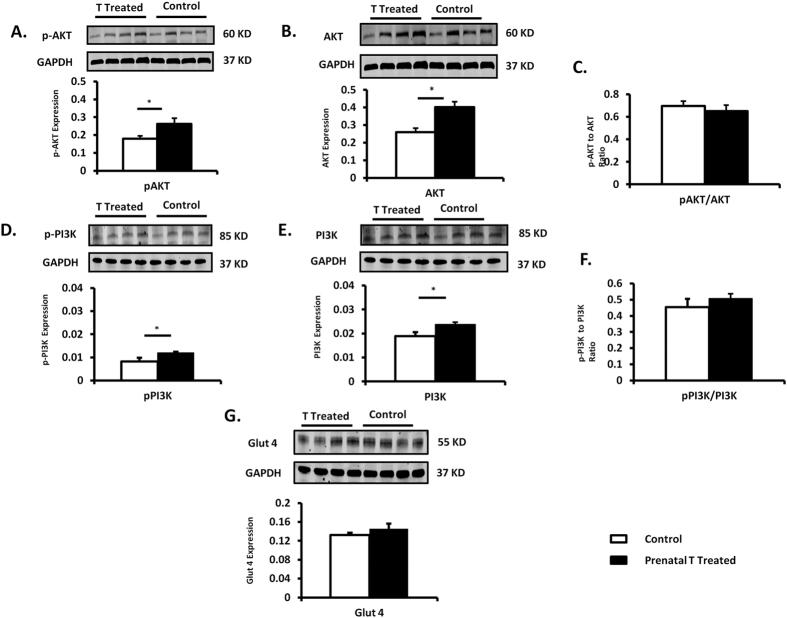
Protein expression (Mean ± SEM) of total and phosphorylated proteins involved in insulin signaling pathway (n = 7–8 per group). Two tail student t-test was used to test difference between T-treated and control and * assigned to p < 0.05. The Cohen’s effect size analysis revealed large differences between T-treated and control group for PI3K, AKT total and phosphorylated proteins involved in insulin signaling pathways (cohen’s d: PI3K = 1.68, pPI3K 2.53; AKT = 1.83, pAKT = 1.14) and small differences in GLUT 4 (cohen’s d: 0.57).

Under basal conditions, protein expression of total PI3K (p85 subunit) in the left ventricle was significantly different (p = 0.01) between prenatally T-treated (0.023 ± 0.0) and control females (0.019 ± 0.001); and pPI3K was also increased (p = 0.0008) in prenatally T-treated (0.01 ± 0.0005) compared to control (0.008 ± 0.0006). Ratio of pPI3K/PI3K was not statistically significant between groups (prenatal T-treated: 0.5 ± 0.03 vs control: 0.45 ± 0.05, p = 0.55). Total AKT (prenatal T: 0.40 ± 0.03 vs control 0.26 ± 0.02; p = 0.007) and pAKT (prenatal T: 0.26 ± 0.03 vs control: 0.18 ± 0.02; p = 0.028) were significantly increased in prenatally T-treated females compared to control. Ratio of pAKT/AKT (prenatal T-treated: 0.65 ± 0.05; vs control: 0.69 ± 0.04) between the two groups was not statistically different (p = 0.57). We did not see any difference in Glucose transporter 4 (GLUT 4) protein levels between prenatal T-treated vs. control (p = 0.33, Fig. 2).

### Mediators of left ventricular hypertrophy (LVH) and stress

**Figure 3:**
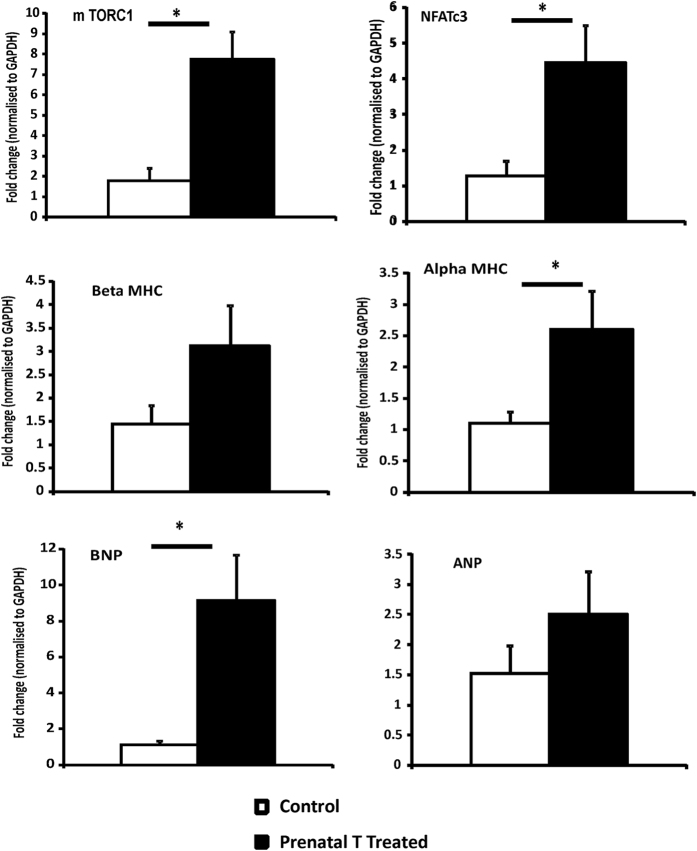
Gene expression (Mean ± SEM) of proteins involved in cardiac hypertrophy and stress measured by qRT-PCR in left ventricle tissue obtained at 2 year of age (n = 6–8 per group). Two tail student t-test was used to test difference between T-treated and control and * assigned to p < 0.05 and ^#^ for p value 0.06. The Cohen’s effect size analysis revealed medium to large differences between T-treated and control group for genes involved in insulin signaling pathways (Cohen’s d: mTORC1 = 1.89, αMHC = 1.20, ßMHC = 0.89, mTORC1 = 1.89, BNP = 1.34, ANP = 0.61, NFATc3 = 1.46). Abbreviations: mTORC1 Mammalian target of rapamycin complex 1, MHC myosin heavy chain, BNP brain naturetic peptide, ANP atrial naturetic peptide, NFATc3 nuclear factor of activated T cells.

**Figure 4:**
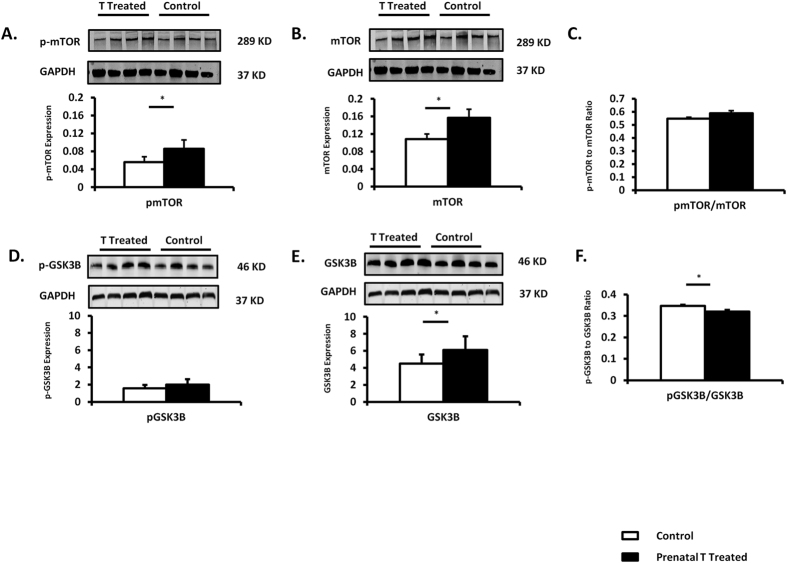
Protein expression (Mean ± SEM) of total and phosphorylated proteins for mTOR and GSK 3 beta (n = 7–8). Two tail student t-test was used to test difference between T-treated and control and * assigned to p < 0.05. The Cohen’s effect size found large differences between T-treated and control groups for proteins involved in insulin signaling pathways (mTOR = 1.21, pmTOR = 1.82; GSK 3 beta = 1.12, pGSK3beta = 0.8).

A 6.1 fold increase in mTORC1, marker of hypertrophic growth downstream from the insulin-signaling pathway that has shown to be activated by T *in-vitro*^25^,^27^ gene expression was found in the left ventricle of prenatally T-treated females compared to control (p = 0.002) (Fig. 3) and its protein expression was also increased in the left ventricle (total mTOR: prenatal T 0.16 ± 0.02 vs control 0.11 ± 0.01, p = 0.04; p mTOR: prenatal T: 0.09 ± 0.008 vs. control 0.056 ± 0.004; p = 0.01) (Fig. 4). GSK3ß, an important downstream target of AKT in the heart, which contributes to suppression of hypertrophic growth^28^, did differ between the two groups. Total GSK3 beta was significantly increased (p = 0.04) in prenatally T-treated females (6.1 ± 0.57) compared to control (4.5 ± 0.4); whereas p-GSK 3 beta was not different (p = 0.16) between prenatally T-treated (1.99 ± 0.22) and control (1.56 ± 0.15) groups. Ratio of p-GSK 3 beta/GSK3beta was significantly lower (p = 0.002) in prenatally T-treated females (0.32 ± 0.008) compared to control (0.35 ± 0.05) (Fig. 4).

Prenatal T treatment significantly upregulated other markers of cardiac hypertrophy including nuclear factor of activated T cells (NFATc3) a well-known marker of cardiac hypertrophy^29^. Prenatal T increased gene expression of NFATc3 by 3.5 fold above control (p = 0.02, Fig. 3). Measures of BNP and ANP, markers of cardiac hypertrophy that are released in response to cardiac wall strain, showed that BNP was elevated by 8.2 fold in the prenatally T-treated group relative to controls (p = 0.01, Fig. 3), while ANP levels did not differ (fold change 1.64: p = 0.28) between groups.

αMHC, the predominant myofilament protein in rodents, shown to be altered in rodent models of cardiac hypertrophy^30^,^31^,^32^, was elevated by 2.3 fold (p = 0.046) in the prenatally T-treated group compared to controls (Fig. 3). In contrast ßMHC the predominant myofilament protein in adult human left ventricle^33^ did not differ significantly (fold change 2.2: p = 0.10) between the 2 groups.

### Steroid receptor expression

**Figure 5:**
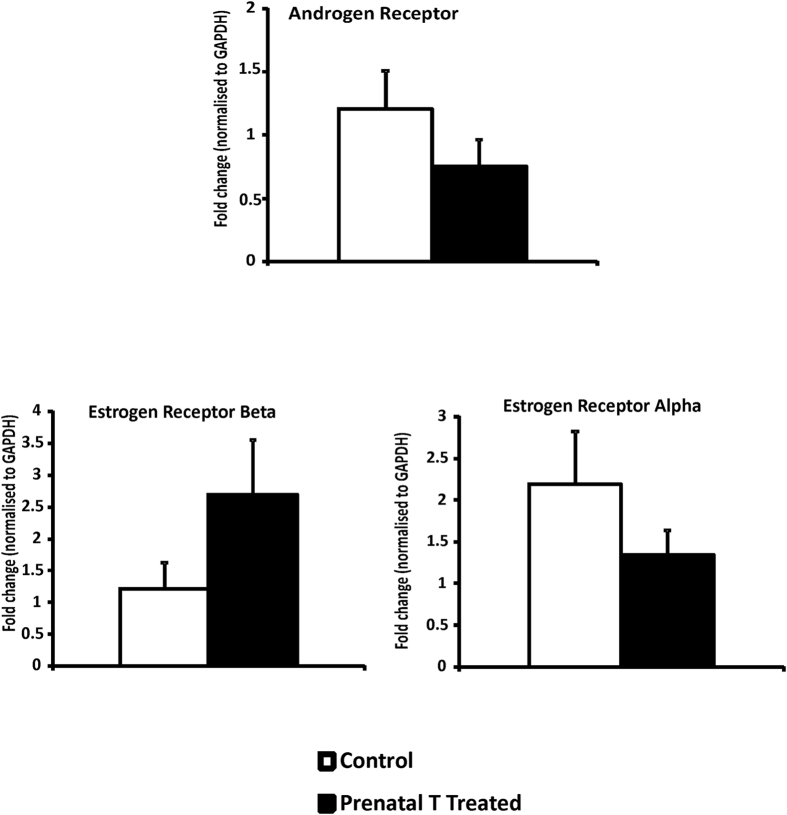
Gene expression (Mean ± SEM) of androgen and estrogen receptor measured by qRT-PCR in left ventricle tissue obtained at 2 years of age (n = 7–8 per group). The Cohen’s effect size found small differences between T-treated and control groups in gene expression of androgen and estrogen receptors (AR = 0.2, ER alpha 0.26 and ER beta 0.77) Abbreviations: AR Androgen receptor, ER estrogen receptor.

Although androgen and estrogen receptors were expressed in left ventricular tissue of sheep, prenatal T treatment did not significantly alter the gene expression of androgen receptor or estrogen receptor beta or alpha (AR fold change 0.88 p = 0.40, ER beta fold change 2.2: p = 0.17, ER alpha fold change 1.5: p = 0.64, Fig. 5).

### Histology results

**Figure 6:**
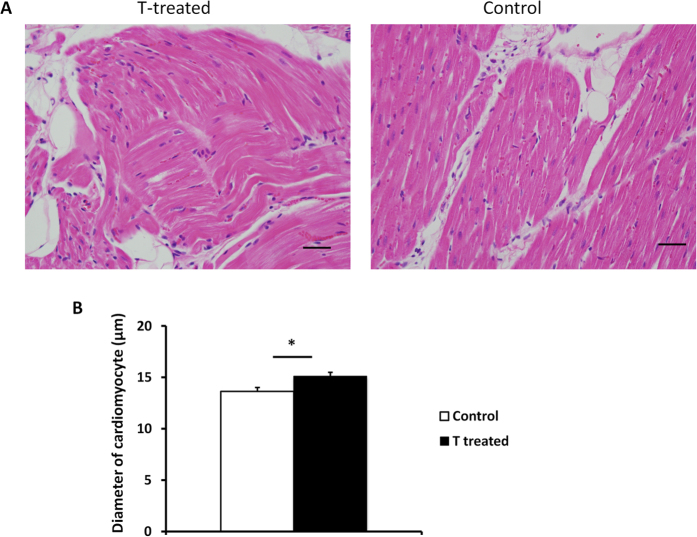
(**A**) Representative histological sections of H&E stained sections from left ventricle tissue from two-year-old prenatal T-treated sheep (A) and control (B). (**B**) Effect of prenatal T treatment on the mean diameter of cardiomyocytes. * Denotes p- value < 0.05. The Cohen’s effect size found large differences between T-treated and control groups with respect to cardiomyocyte width (Cohen’s d = 1.473).

5 out of the 8 prenatally T-treated females had focal myocardial disarray in left ventricle on both H&E stain and Masson staining compared to controls that had no evidence of myocardial disarray (Fig. 6a). Prenatal T treatment increased cardiomyocyte size compared to control (T-treated 15.1 ± 0.4 μm vs 13.6 ± 0.4 μm, p = 0.02) (Fig. 6b).

## Discussion

Findings from the current study, namely increased expression of genes and proteins involved in left ventricular hypertrophy and stress, together with histological evidence of focal myocardial disarray and an increase in cardiomyocyte size, suggest that prenatal exposure to excess T likely alters the trajectory of left ventricular tissue differentiation in the female sheep. This differentiation appears to involve upregulation of proteins involved in insulin signaling and downstream upregulation of mTOR, a known marker of hypertrophic growth. To our knowledge this is the first study to show perturbed molecular signaling in the left ventricle in a well-established sheep model of PCOS that also manifests IUGR during fetal development^18^,^22^. These perturbations in molecular signaling could lead to increased susceptibility of injury to the heart.

Increased left ventricular mass has been shown to predict increased cardiovascular morbidity and mortality^34^,^35^. Patients with hypertension and high plasma insulin are more likely to develop LVH^36^,^37^. Systemic insulin resistance has also been linked to LVH^38^,^39^,^40^. Consistent with this premise, Cohen’s effect size analysis revealed a modest increase in fasting insulin and insulin/glucose ratio in the prenatal T-treated females. The potential mechanism by which insulin resistance is linked to LVH is through metabolic dysregulation (hyperglycemia and hyperinsulinemia) that leads to altered myocardial protein degradation, altered matrix remodeling, increased cellular lipids and altered vascular compliance, eventually leading to LVH and remodeling^40^. Findings to date provide evidence that prenatal T-treatment leads to insulin resistance in insulin target tissues, hyperinsulinemia and hypertension^15^,^18^,^26^, which are all risk factors for development of LVH. Alternatively, some of the altered molecular signaling may occur *in-utero* secondary to the direct effect of T excess on the cardiac tissue and further accentuated by the perturbed systemic environment postnatally. Although prenatal T treatment did not lead to statistically significant increases in weight of the left ventricle the molecular changes and histological changes suggest that these early perturbation may eventually lead to such changes in LVH over time.

mTORC1 is one of the key regulators of protein synthesis in the cardiomyocyte and activation of this has been shown to lead to cardiac hypertrophy^41^,^42^. It can be activated by growth factors including insulin through its signaling pathway and also by T^25^,^43^. Activation of proteins downstream from the insulin receptor (PI3K/AKT/mTORC1) has been linked to “physiological hypertrophy” in the heart but there is also evidence for “pathological” hypertrophy with chronic activation of this pathway^44^,^45^,^46^,^47^,^48^ leading to disruption of coordinated tissue growth and angiogenesis^48^. Findings from this study showing increased expression of mTORC1, PI3K, and AKT suggests that prenatal T excess may underlie activation of cardiac insulin signaling pathway resulting in activation of mTORC1 culminating ultimately to cardiac hypertrophy. Increase in gene expression of BNP and NFATc3 both markers of pathological hypertrophy are suggestive of a maladaptive hypertrophy.

Because prenatal T excess leads to maternal hyperinsulinemia^49^ and postnatal insulin resistance^18^ in the offspring, it is likely that maternal hyperinsulinemia could also be a major player in programming prenatal T-induced cardiac hypertrophy in offspring. For instance, increase in PI3K/AKT/mTORC1 signaling is seen in newborns with congenital hyperinsulinemia^50^,^51^. Similarly, fetal hyperinsulinemia due to maternal obesity has been shown to lead to cardiac programming defects in the offspring with increase in markers of left ventricular hypertrophy and increase in AKT and also mTOR^52^.

While T can lead to cardiac hypertrophy indirectly via programming of hyperinsulinemia, the potential for direct effects of androgen on cardiac hypertrophy cannot be ignored. These animals are functionally hyperandrogenic and manifest increased expression of AR at the level of the hypothalamus^53^, pituitary (Cordoso and Padmanabhan, unpublished) and ovary^54^. Cardiomyopathy has been seen in newborns with congenital adrenal hyperplasia^55^, a condition where the fetus is exposed to excess endogenous androgen. Androgen and estrogen receptors are present in cardiac tissue both in the cytosol and the nucleus^19^. Sex hormone binding to their receptors have been found to lead to both genomic and non-genomic effects in the heart, and one mechanism is through activation of the PI3K/AKT pathway^19^ that leads to downstream activation of mTORC1 resulting in increased protein synthesis and eventually cardiac hypertrophy^24^. While androgen receptors are expressed in the left ventricle to facilitate direct effects of T, absence of changes in AR expression in prenatal T-treated females suggests that any direct effects of T may be mediated via the increase in ligand (T treatment) rather than the receptor.

T has been found to increase cardiomyocyte glucose uptake by augmenting GLUT4 translocation^56^ and although an increase in GLUT 4 protein expression was not seen in the left ventricle under basal condition, the increase in expression of insulin signaling proteins including IRS-1, PI3K and AKT is suggestive of increase in glucose metabolism within the left ventricle. Free fatty acids are the primary fuel for energy production in cardiac tissue but during fetal life and during times of stress, the heart does revert to glucose as its primary source of fuel^57^. As such, an increase in the gene expression of proteins involved in glucose uptake in prenatal T females may signify an adaptive response to increased metabolic demand on the heart since we also see an increase in the expression of BNP, a cardiac stress marker. The elevated mTORC1 expression suggests that T could lead to cardiac hypertrophy through increases in mTORC1 as suggested previously^25^.

Offspring that have experienced an adverse intrauterine environment during a critical period of development culminating in IUGR, such as that evident in prenatal T-treated sheep^16^,^58^ have alterations at the molecular level that builds a platform for increased susceptibility to cardiovascular disease later in life^59^,^60^. It is not clear whether the molecular changes seen in prenatally T-treated sheep involve similar mechanisms that lead to adverse cardiac programming in other models of IUGR. For instance, 50% nutrient restriction in sheep (a model of IUGR) leads to upregulation of a variety of genes in the fetal heart, some of which are involved with cardiac hypertrophy and metabolism^61^,^62^ and altered fatty acid content of the cardiomyocytes^62^. Functional changes in the heart have also been observed in offspring of rats treated with low protein diet^63^. Glucocorticoid excess prenatally has also been shown to lead to IUGR accompanied with hypertension and insulin resistance as seen with prenatal T excess^64^,^65^. Direct molecular effects on the myocardium have been seen with *in-utero* exposure to excess glucocorticoids including increased expression of cardiac glucose transporter 1 (one of the main glucose transporter in the heart)^66^ and upregulation in AKT/PKB pathway^66^, similar to what we see in our model.

From a translational standpoint, these findings may be of relevance to female offspring of women with PCOS. It has been shown that androgen and insulin levels are elevated in pregnant women with PCOS^67^,^68^ and there is some evidence with Spanish cohorts that they give birth to babies with reduced birth weight^69^. The female offspring of PCOS mothers have early metabolic derangement^70^ similar to that seen in our prenatal T model^18^, suggesting that as in the prenatally T-treated sheep model, presence of hyperandrogenism in PCOS women has the potential to adversely program several organ systems including the cardiovascular system in their offspring.

In summary, findings from this study provide evidence for cardiac reprogramming in a sheep model of PCOS. Further studies delineating the functional consequences of such changes are vital and will further our understanding the effect of prenatal T excess on cardiac health and assist in developing new treatment strategies towards preventing cardiac susceptibility to prenatal insults.

## Material and Methods

Suffolk female lambs born to dams that received twice weekly intramuscular injections of 100 mg of T propionate (1.2 mg/kg; Sigma-Aldrich Corp., St. Louis, MO) in cottonseed oil from days 30 to 90 of pregnancy were used in the study. Control breeders were injected with the vehicle for the same duration. The concentrations of T achieved in maternal circulation with this mode of T delivery are in the range of adult males^71^. The concentrations of T achieved in female fetuses with this mode of T delivery are also within range seen in the control fetal males^71^. Mothers were maintained on pasture and supplemented with hay and concentrated pelleted food to meet the increased needs of the pregnant sheep. Breeder sheep diet ranged from 2.6–2.9 (Mcal/kg) of digestible energy based on NRC requirements throughout pregnancy as detailed in a previous publication^27^. All lambs were born by normal delivery and after full length gestation. After weaning (8 weeks of age), lambs were placed on an adjusted diet of commercial feed pellets (Shur-Gain, Elma, NY). A reduction in crude protein was implemented when they reached 40 kg of body weight as previously described^72^. Lambs were maintained with their mothers under natural photoperiod and with free access to water. All procedures for management and experimental methodologies were approved by the Institutional Animal Care and Use Committee of the University of Michigan, which are consistent with the National Research Council’s Guide for the Care and Use of Laboratory Animals.

At approximately 2 years of age, to avoid influence from differing levels of endogenous steroids, all animals were ovariectomized and implanted with a 1-cm-long SILASTIC capsule (inner diameter, 3.35 mm and outer diameter, 4.65 mm; Dow Corning Corp., Midland, MI) filled with 17β estradiol (estradiol; Sigma-Aldrich, St. Louis, MO, USA). Approximately 1 month later, two controlled internal drug release progesterone implants (InterAG, Hamilton, Waikato, New Zealand) were inserted subcutaneously into each animal. Progesterone implants were removed 14 days later and all animals received four additional 3-cm-long estradiol implants to achieve ovarian steroid levels found during the late follicular phase. Animals were euthanized ~20 hours after insertion of the estradiol implants during the presumptive late follicular phase of the cycle by administration of barbiturate overdose (Fatal Plus, Vortech Pharmaceuticals, Dearborn, MI). Hearts were harvested and portions of right and left ventricle were both quickly frozen and stored at −80 °C until processing or formalin fixed for histological processing. Plasma insulin and glucose were measured prior to euthanasia after 48 hours fast. Concentrations of insulin were determined by radioimmunoassay (insulin RIA Kit, MP Biomedicals). Assay sensitivity of insulin assay was 3.0 μU/L. Glucose concentrations were assessed by the glucose oxidase method (Pointe Scientific Inc.). Inter- and intra-assay CV for both the insulin and glucose assays were <10%.

### Isolation of RNA and Real Time Polymerase Chain Reaction

RNA from left ventricular tissue (n = 8 prenatal T-treated and n = 7 control) was isolated using TRIzol reagent (Invitrogen, Carlsbad, CA). Purification of RNA was done with deoxyribonuclease treatment. Purity of the RNA for each sample was validated by determining its RNA Integrity Number (RIN). Concentration was determined using a nanodrop spectrophotometer. First strand cDNA was synthesized from RNA using Superscript III first strand synthesis system (Invitrogen Cat. No: 18080-051). cDNA was prepared from 1 μg total RNA. Concentrations of cDNA over 1000 fold range was amplified using each target gene and GAPDH specific primers to determine the final concentration at which each primer sequence for both housekeeping gene and gene of interest were equally and >90% efficient. Gene specific primers were designed using primer select software and are detailed in supplemental material Table 1.

The SYBR Green real time PCR assay was performed using the cDNA generated. Negative reverse transcriptase control was run for each sample to rule out genomic DNA contamination of isolated RNA. Quantitative RT PCR reactions were run on Applied Biosystem’s 7200 Real Time PCR instrument, and were performed in triplicate. Results of the assay were quantified using the cycle threshold (CT) values of each sample. The CT values of each gene of interest were compared against those of the housekeeping gene, glyceraldehyde 3-phosphate dehydrogenase (GAPDH). Only values less than 35 were considered positive. The results of each assay were verified by replicating the outcome in a second test.

### Western Blot

Sheep left ventricular tissue was crushed under liquid nitrogen and protein lysate was generated using lysis buffer (Triton 1%, sodium vanadate (1 nM), sodium fluoride (50 mM) and sodium pyrophosphate 10 mM and protease inhibitor cocktail 10 mM). Each sample was normalized to contain 2 μg/μl by adding lysis buffer, Laemelli buffer (Bio-rad, Cat. no: 161-0737) and Beta-mercaptoethanol to the lysate. 15 μl of the sample was loaded onto 4–12% Bis-Tris gels (Life Technologies Cat. No: NP0321BOX) and gel electrophoresis was performed using the XCell Sure Lock chamber (Invitrogen, Carlsbad, CA). The proteins were then transferred to Optitran Reinforced Nitrocellulose (Whatman, Cat. no: 10439396) at 100 Volts for one hour. The blots were blocked for one hour in Odyssey Blocking Buffer (Li-Cor, Lincoln, NE. Cat. No: 927-40000). Primary antibody (polyclonal rabbit) as detailed in supplemental material Table 2, for the AKT, p-AKT (serine 473), p85 subunit of PI3Kinase and p-PI3K and insulin-inducible, glycogen synthase kinase-3ß (GSK3ß), glucose transporter type 4 (GLUT 4) were obtained from Cell Signaling Technology (Danvers, MA) and the housekeeping gene GAPDH (mouse monoclonal) antibody was obtained from Sigma-Aldrich (St. Louis, MO). The primary antibodies were left to incubate overnight at 4 °C. Blots were then washed and incubated with Li-COR Secondary antibody (1:10000) of Donkey anti- Rabbit (680RD) and Donkey anti- Mouse (800 CW). Western blots were developed with the Odyssey infrared imaging system (LI-COR Biosciences, Lincoln, NE). Band intensities were quantified using the Odyssey software (LI-COR Biosciences) and normalized to GAPDH.

### Histopathological analysis

Left ventricular tissues were fixed in formaldehyde in PBS pH 7.4. The tissue was then dehydrated through serial alcohols, cleared in xylenes, and embedded in paraffin. Paraffin sections with a thickness of 5 μm were cut and stained with hematoxylin and eosin (H&E) for gross appearance analysis. Periodic acid-Schiff counterstained with hematoxylin (PAS-H) was used to facilitate quantification of cardiomyocyte size by our Investigative Histopathology laboratory at Michigan State University. Histological sections were reviewed in random order by two independent veterinary pathologists that were blinded to treatment groups.

### Histomorphometry

Following previously established protocols^73^,^74^ left ventricular wall cardiomyocyte mean diameter of each sample was determined by measuring the short axis of 100 cardiomyocytes in nucleated transverse section with nearly circular capillary profiles in 10 randomly selected microscopic fields at 400X magnification. Measurements were obtained with CellSens software.

### Statistical analysis

The cardiomyocyte diameters between treatment groups were compared using two tailed student t-test. To determine changes in gene expression, ΔCT formula (Ct for target gene-Ct for GADH gene) was used to normalize each target gene Ct value for control and T-treated animals. Relative gene expressions were determined using formulae ΔΔCt = ΔCt_T-treated_ − ΔCt_control_ and relative target mRNA level was analyzed following the 2^−ΔΔCt^ method as previously described^75^. Results are presented as mean 2^−ΔΔCt^ ± SEM. For each gene, the difference between control and prenatally T-treated females was determined using two-tailed Student’s *t-test*. Student two-tailed t-test was also used to compare differences between protein expressions of control vs. prenatally T-treated females after normalizing the protein of interest to GAPDH. P-values are reported for each test. In addition, to complement findings from the t-test, Cohen’s effect size analysis was performed to address the magnitude of changes. This analysis allows comparison of the means between two treatments with respect to the magnitude of difference between them. The computed statistic is Cohen’s d value, and values above 0.2, 0.5, and 0.8 were considered as small, medium, and large effect sizes, respectively^76^ Cohens values for data presented in the figures are provided in the legend.

## Additional Information

**How to cite this article**: Vyas, A. K. *et al*. Prenatal programming: adverse cardiac programming by gestational testosterone excess. *Sci. Rep.* **6**, 28335; doi: 10.1038/srep28335 (2016).

## Supplementary Material

Supplementary Information
