# Supplementary material for: Prenatal programming: adverse cardiac programming by gestational testosterone excess
Source: Sci Rep. 2016 Jun 22;6:28335. doi: 10.1038/srep28335 (PMC4916456; doi:10.1038/srep28335)
Supplement: Supplementary Information [file srep28335-s1.doc]

**Prenatal programming: adverse cardiac programming by gestational testosterone excess**

**Arpita K Vyas^1*^, Vanessa Hoang^1^, Vasantha Padmanabhan^2^, Ebony Gilbreath^3^, Kristy A Mietelka ^4^**

| Gene name | Forward Sequence | Reverse Sequence | Accession Number |
| --- | --- | --- | --- |
| AKT | CGGCTCCCTCTCCTGTTAGG | GGGATTTTCCAGCCAAGAGTACT | AF207873 |
| GAPDH | AAGGTGGTGAAGCAGGTGTCA | TGGTCTTCAGTGTAGCCTAGAATGC | NM_001190390 |
| Insulin receptor substrate 1 (IRS-1) | CGCTCCAGCGAGGATCTAAG | AGGTCCTCTGGCTGCTTCTG | XM_004004977 |
| PI3K | TTGTCCAGCCACCATGATGT | TGAGCAAGAGGCTTTGGAATATT | M93252.1 |
| mTORC1 | ATCACCCTTGCTCTCCGAACTCTC | CCAGCTCCCGGATCTCAAACACCT | NM_001145455 |
| Atrial natriuretic peptide (ANP) | GCTCCTAACGGGCAATTTGTT | CCTCCACTCCAGCCTGATGA | AF037465 |
| brain naturetic peptide (BNP) | GACCCAGAAGTGGCTCTAATGG | CGCATTCCCACTGCA | AF037466 |
| beta-myosin heavy chain (ßMHC) | TCTCCTCCCAGCAGCAATCC | GGTCACTGCTCCATCGTTGC | NP_001116874 |
| alpha-myosin heavy chain (α MHC) | ACATTGCTCACTACAGCCTTGTG | GGTCCAAGGAGAGAAACATGCT | NM_001123398 |
| Androgen Receptor | GCCCCTGACCTGGTTTTCA | TTCGGACACACTGGCTGTACA | KF227907 |
| Estrogen Receptor α | ACTGTGCAGTGTGAATGAC | TATAAAACCAAGCCTCACCT | AY033393.1 |
| Estrogen Receptor ß | GATGTGGGTACCGCCTTGTGC | GGCCAACTTGGTCAGGGACA | AF110817 |
| Nuclear factor of activated T cells –c3 (NFATc3) | CTACTGGTGGCCATCCTGTTGT | AGCTCTTGAGCAGATCGCTGAGAGCACTC | NC019471 |
| Glycogen synthase kinase 3ß (GSK3ß) | AGACAAAGATGGCAGCAAGGTGAC | ACGCAATCGGACTATGTTAC | NM_001129740 |

**Table 1: Primer sequences**

**Table 2: Antibodies**

| **Peptide/protein target** | **Name of Antibody** | **catalog #** | **Species raised in; monoclonal or polyclonal** | **Dilution used** |
| --- | --- | --- | --- | --- |
| PI3K p85 | PI3K p85 Antibody | 4292 | Rabbit, Polyclonal | 1:800 |
| AKT | AKT Antibody | 9272 | Rabbit, Polyclonal | 1:800 |
| GSK-3β | GSK-3β (27C10) Antibody | 9315 | Rabbit, Polyclonal | 1:800 |
| mTOR | mTOR (7C10) Antibody | 2983 | Rabbit, Polyclonal | 1:800 |
| P-AKT (Ser473) | Phospho-AKT (Ser473) Antibody | 9271 | Rabbit, Polyclonal | 1:800 |
| P-PI3K Kinase p85 | Phospho-PI3K Kinase p85 (Tyr458)/p55(Tyr199) Antibody | 4228 | Rabbit, Polyclonal | 1:800 |
| P-GSK-3β (Ser9) | Phospho- GSK-3β (Ser9) Antibody | 9323 | Rabbit, Polyclonal | 1:800 |
| P-mTOR (Ser2448) | Phospho-mTOR (Ser2448) Antibody | 2971 | Rabbit, Polyclonal | 1:800 |
| GLUT 4 | Anti- GLUT4 Antibody | ab654 | Rabbit, Polyclonal | 1:1000 |
| GAPDH | Anti-GAPDH Antibody | G8795 | Mouse, Monoclonal | 1:5000 |
